# Innovative inbuilt moving bed biofilm reactor for nitrogen removal applied in household aquarium

**DOI:** 10.3389/fmicb.2024.1373119

**Published:** 2024-04-09

**Authors:** Xiaolin Zhou, Haicheng Liu, Xing Fan, Xinhao Xu, Yuan Gao, Xuejun Bi, Lihua Cheng, Shujuan Huang, Fangchao Zhao, Tang Yang

**Affiliations:** State and Local Joint Engineering Research Centre of Urban Wastewater Treatment and Reclamation, Qingdao University of Technology, Qingdao, China

**Keywords:** ammonia, nitrification, wastewater treatment, moving bed biofilm reactor, biofilm

## Abstract

An innovative inbuilt moving bed biofilm reactor (MBBR) was created to protect fish from nitrogen in a household aquarium. During the 90 experimental days, the ammonia nitrogen (NH_4_^+^-N) concentration in the aquarium with the inbuilt MBBR was always below 0.5 mg/L, which would not threaten the fish. Concurrently, nitrite and nitrate nitrogen concentrations were always below 0.05 mg/L and 4.5 mg/L, respectively. However, the blank contrast aquarium accumulated 1.985 mg/L NH_4_^+^-N on the 16th day, which caused the fish to die. The suspended biofilms could achieve the specific NH_4_^+^-N removal rate of 45.43 g/m^3^/d. Biofilms presented sparsely with filamentous structures and showed certain degrees of roughness. The bacterial communities of the suspended biofilms and the sediment were statistically different (*p* < 0.05), reflected in denitrifying and nitrifying bacteria. In particular, the relative abundance of *Nitrospira* reached 1.4%, while the genus was barely found in sediments. The suspended biofilms showed potentials for nitrification function with the predicted sequence numbers of ammonia monooxygenase [1.14.99.39] and hydroxylamine dehydrogenase [EC:1.7.2.6] of 220 and 221, while the values of the sediment were only 5 and 1. This study created an efficient NH_4_^+^-N removal inbuilt MBBR for household aquariums and explored its mechanism to afford a basis for its utilization.

## 1 Introduction

Keeping ornamental fish as pets in an aquarium is increasingly popular in households (Perry, 2023). However, fish will produce ammonia in the aquarium, which is one of the most common water quality problems affecting the ornamental fish to die (Helen Roberts et al., 2008). The ammonia tolerance for the warmwater and coldwater fishes was reported to be just 3 and 1 mg/L, respectively (Yildiz et al., 2017). Higher concentrations of ammonia would cause the fish to die. This happens especially when the aquarium is first set up. Therefore, how to protect the fish from ammonia would be the key to establish an aquarium.

At present, the methods widely used in household aquariums to remove ammonia nitrogen are the following: (i) change the water several times in small amounts; (ii) add nitrifying bacteria liquid inoculant or tablet; and (iii) establish an external filtration system for wastewater treatment and circulation. However, the first method requires a lot of workforces and wastes a lot of water. Thus, it has apparent defects. Although the second method is easy to use and quick to effect, it requires repeated dosing because nitrifying bacteria are readily eaten by fish, leading to high costs. The third method needs to occupy space to place the filtration system, which will destroy the aquarium’s beauty. Moreover, the filter material needs to be cleaned or replaced regularly. In conclusion, a device that is small, convenient, and beautiful for nitrogen removal in the household aquarium is required.

Moving bed biofilm reactor (MBBR) has turned out to be an efficient wastewater treatment technology, and it has the advantage of high pollutant removal rates (Guo et al., 2010; Ashkanani et al., 2019). It uses specially designed suspended carriers for biofilm attachment and enrichment. The biofilms are always maintained in suspension and throughout the reactor by turbulent energy imparted by aeration or other disturbing forces (Water Environment Federation, 2010). This technology has been extensively used in urban sewage treatment (Zhou et al., 2022b). It presented a strong ammonia nitrogen removal ability (Zhou et al., 2023). The introduction of MBBR to the household aquarium is expected to become one of the most effective methods for nitrogen removal. Firstly, MBBR is a compact process (Ødegaard, 2016), so it would be very small. Secondly, the biofilms attached to the suspended carriers in MBBR could renew automatically due to hydraulic shear, and the shed biofilms could be used as bait for the fish. So there is no need to replace or clean the carriers, which would be convenient. Finally, the suspended carriers could be colorful, so the inbuilt MBBR could add the aquarium’s ornamental features. However, is it feasible to apply MBBR to protect fish from ammonia in a household aquarium? It is rarely reported.

In this study, an innovative inbuilt MBBR was designed and established in a household aquarium. The details of ammonia nitrogen (NH_4_^+^-N), nitrite nitrogen (NO_2_^−^-N), nitrate nitrogen (NO_3_^−^-N) and chemical oxygen demand (COD) concentrations throughout experimental periods in the aquarium were presented. In addition, the nitrification performance of the biofilms was tested, and the biofilm morphology was observed. Moreover, high-throughput sequencing technology was used to investigate bacterial community in the inbuilt MBBR, and the potential functions for nitrogen removal were also analyzed simultaneously. This study aimed to establish a high-efficiency ammonia nitrogen removal device in household aquariums with the benefit of being small, convenient, and beautiful and provide a scientific basis for its design and application.

## 2 Materials and methods

### 2.1 Set-up of inbuilt MBBR in a household aquarium

**Figure 1:**
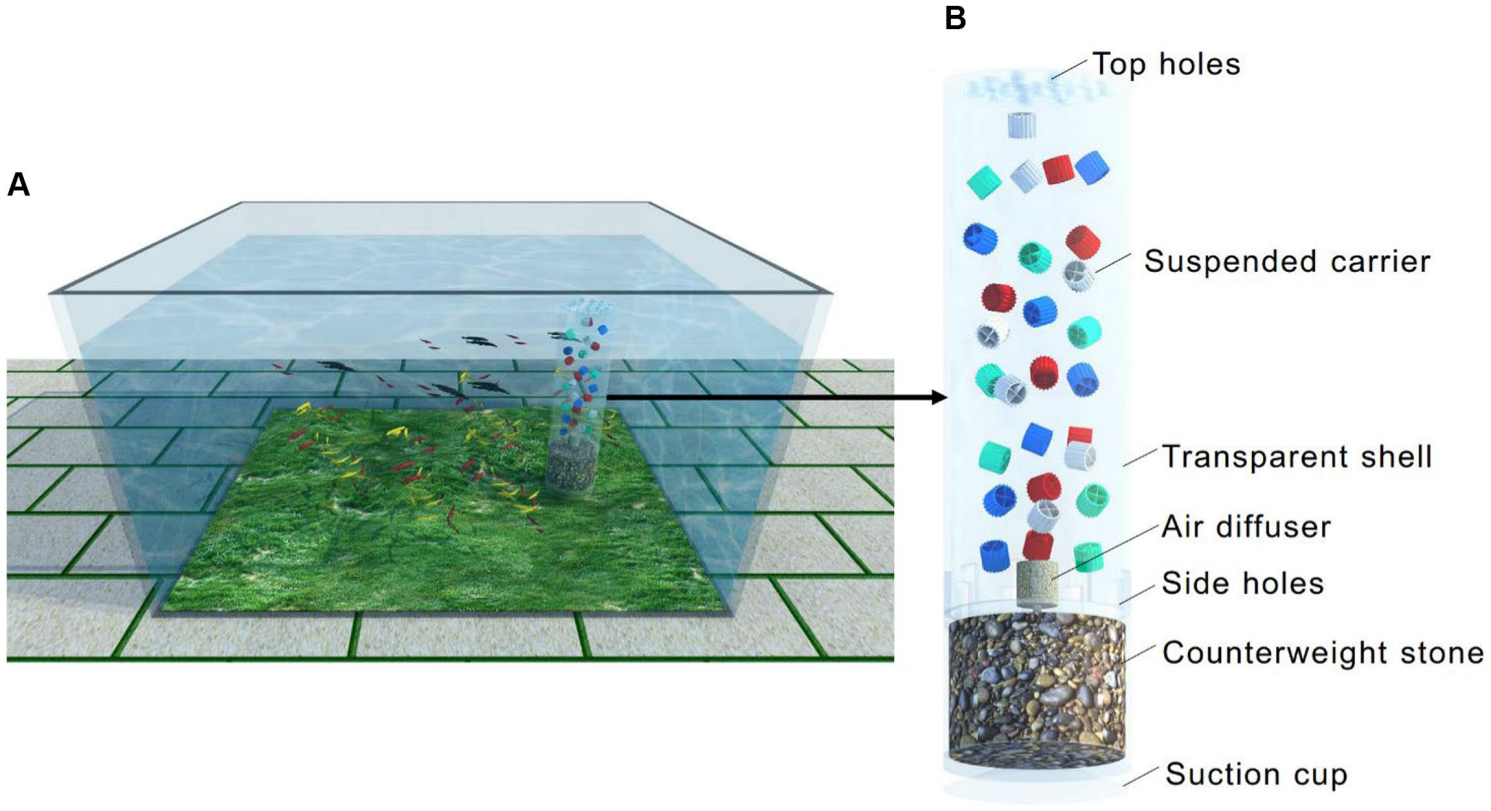
Schematic of the inbuilt MBBR in the household aquarium: application scenarios **(A)** and details of the composition **(B)**.

A schematic diagram of an inbuilt MBBR in the household aquarium is shown in Figure 1. As shown in Figure 1A, the inbuilt MBBR was immersed in the aquarium. The device’s length, width and heigh were 50 cm, 28 cm, and 38 cm, respectively. The depth of water was 35 cm. There were 88 guppies with a body length of 2–4 cm in the aquarium. As illustrated in Figure 1B, the shell of the equipment is a transparent cylindrical plastic, including an upper layer and a lower layer. The upper layer is the microbial reaction zone with a diameter of 6 cm and a height of 20 cm and was filled with plastic carriers with a fraction of 50%. For esthetic reasons, the carriers came in four colors, including equal amounts of white (without dyestuff), red (with red dyestuff), green (with green dyestuff), and blue (with blue dyestuff). The carriers in the reactor were the type of K1 with a depth of 7 mm, a diameter of 9 mm, and a specific surface area of 500 m^2^/m^3^ (Water Environment Federation, 2010). It should be noted that the density of carriers was close to that of water (0.94–0.97 g/cm^3^). The air diffuser was also inside the reactor, providing air to the water, and it was connected to the aeration pump with a nominal voltage of 10 W through a hose. The carriers were held in suspension throughout the reactor by turbulent energy imparted by aeration. There were eight side rectangular holes evenly arranged on the bottom of the upper layer shell with a width of 5 mm and a length of 7 mm. There were also eight round holes evenly arranged on the top of the shell with a diameter of 8 mm. Under the action of gas lifting, water in the aquarium flowed into the reactor through the rectangular holes on the bottom side and then was discharged through the upper round holes with bubbles. There was a suction cup at the bottom of the equipment to attach it to the bottom of the aquarium. At the same time, the lower layer was filled with colorful stones to make the equipment more stable in the water. The inbuilt equipment in the aquarium seemed beautiful due to the colorful suspended carriers dancing, increasing the ornamental of the household aquarium.

### 2.2 Experimental operation

At the beginning of the experimental operation, all the water in the aquarium was tap water after exposure to the sun for 2 h, and simultaneously the fish food was first fed with an amount of ~3.0 g. After that, it was provided once a week. The temperature of the water in the aquarium was always ~25°C. The composition analysis of the fish food is shown in Table 1. During the operating time, the water samples were taken every day. COD, NH_4_^+^-N, NO_2_^−^-N and NO_3_^−^-N concentrations were tested using the standard methods (The State Environmental Protection Administration of China, 2022). The blank contrast aquarium was operated under the same condition as the experimental aquarium, except that there was no inbuilt equipment. The experimental aquarium was operated for 3 months without changing water.

**Table 1:** Composition analysis of the fish food.

Crude protein	Crude fat	Crude fiber	Crude ash	Moisture	Phosphorus	Calcium	Lysine
≥35%	≥ 2.0%	≤8.0%	≤15%	≤10%	≥0.8%	≤2.5%	≥1.0%

### 2.3 Nitrification performance test

After the experimental operation, the bio-carrier samples in the inbuilt MBBR were taken to a 1 L container filled with distilled water with a fraction of 28%. Ammonium chloride was added to the beaker until the NH_4_^+^-N concentration of the water was 15 mg/L. Aeration was provided in the breaker to enable complete fluidization of the suspended bio-carriers and the dissolved oxygen (DO) concentration was above 6.0 mg/L. The water samples were taken at regular intervals, and NH_4_^+^-N, NO_2_^−^-N and NO_3_^−^-N concentrations were also tested.

### 2.4 Biofilm morphology

After the experimental operation, some bio-carriers that were still wet were scanned by the type microscope (OLYMPUS SZX10 microscope) to observe their physical features. The new carriers were monitored as blank groups.

At the same time, the biofilms attached to the carriers, which are called suspended biofilms hereafter, were observed by the scanning electron microscope (SEM; FEI Quanta FEG 250). The bio-carrier samples were cut to the appropriate size (about 5 mm × 5 mm), glued to the sample table with conductive tape, and tested after spraying gold. The acceleration voltage was adjusted to 8 kV for shooting, and the magnification was 2,000. The sediment in the aquarium was also scanned.

The cut bio-carrier samples were smoothed out, and the three-dimensional morphology was measured by laser scanning confocal microscopy (LSCM; Leica DCM8). And then, the shooting image was analyzed by the Leica Fotos App.

### 2.5 Bacterial community

Four carrier samples of white, red, green, and blue were randomly taken after the experimental operation. Then, the biofilms attached to the carriers were scratched off, and the biofilms in the same color carriers were mixed evenly in one biofilm sample. For comparison, sediment samples were taken from three random places at the bottom of the aquarium. The seven samples were commissioned by Majorbio Bio-Pharm Technology Co., Ltd. (Shanghai, China) for genomic DNA extraction and sequencing according to the standard steps (Zhang et al., 2020). The hypervariable region V3–V4 of the bacterial 16S rRNA gene was amplified with the primer pairs presented in 338F and 806R (Zhou et al., 2022a). The raw data were submitted to NCBI and the accession number is PRJNA897105.

The steps of processing of sequencing data were also performed by the standard steps of Majorbio Bio-Pharm Technology Co., Ltd. (Shanghai, China; Zhang et al., 2020). The taxonomy of each operational taxonomic unit (OTU) representative sequence was analyzed against the Silva138 database^1^ using confidence threshold of 0.7. Most of the statistical analysis was performed using the online platform of Majorbio Cloud Platform^2^ (Ren et al., 2022). The heatmap for the relative abundance of the top 30 genera was analyzed by the “pheatmap” package (1.0.8) of R Programming Language (Version 3.3.1). The beta diversity of bacterial communities (OTU level) was analyzed by using principal component analysis (PCA) after a centered log-ratio transformation by the R Programming Language (Version 4.3.2) based on the distance algorithm of Aitchison (Gloor et al., 2017). Simultaneously, the Analysis of Similarities (ANOSIM) was used to test the significance of the difference. The collinear network analysis was performed by Networkx (Version 1.11).

### 2.6 Potential functions analysis

Phylogenetic investigation of communities by reconstruction of unobserved states (PICRUSt) was applied to analyze the potential functions (Zhao et al., 2022). Because of the renewal and elaboration of reference genome databases, the accuracy of PICRUSt2 has grown (Douglas et al., 2020); PICRUSt2 was therefore used in this study. The literatures introduced the detailed operating and analysis procedures (Baquiran et al., 2020; Li et al., 2022). The information on the functional genes for nitrogen removal, involving nitrification and denitrification, was predicted based on the map00910 of KEGG.^3^

## 3 Results and discussion

### 3.1 Overall performance

**Figure 2:**
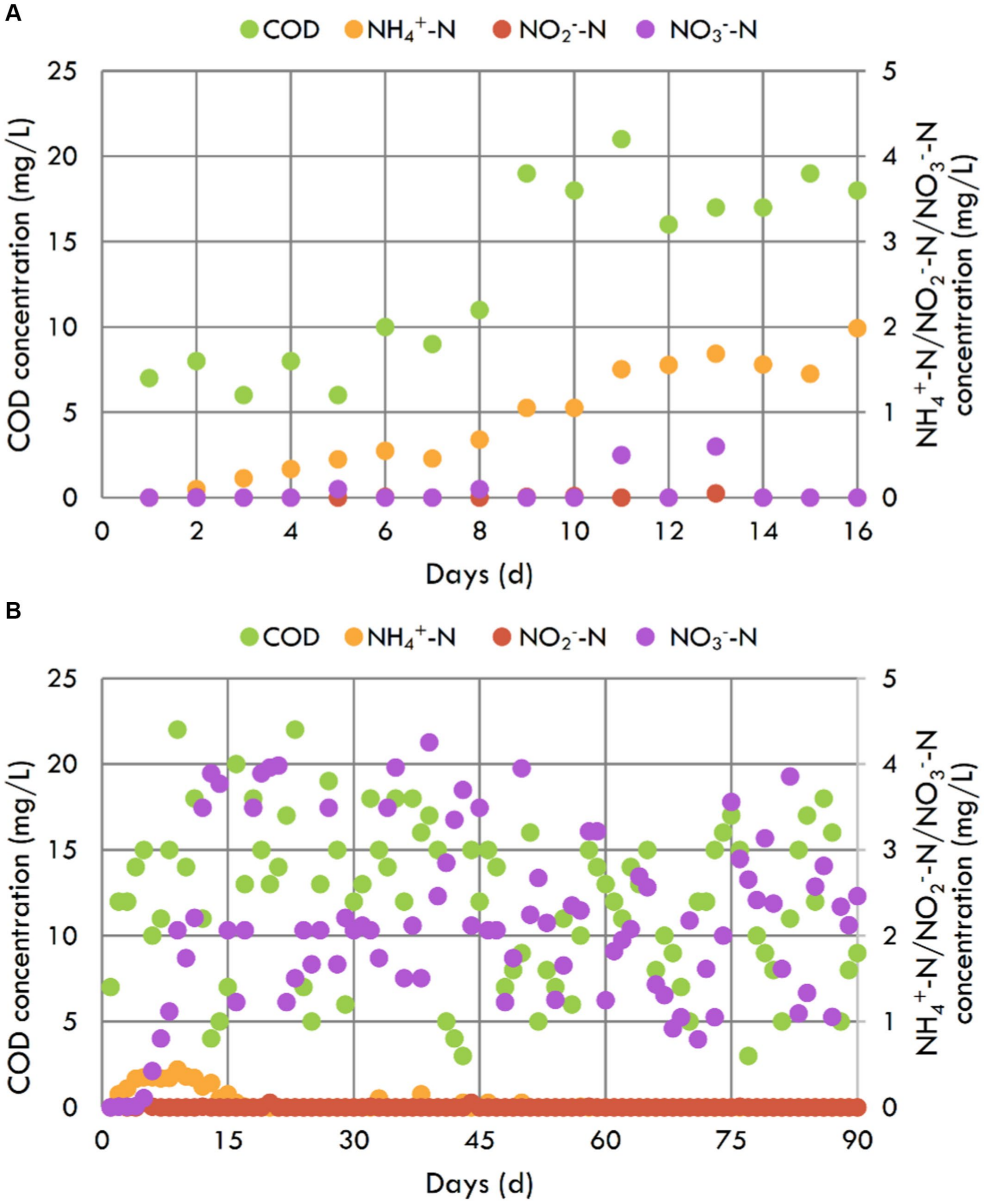
COD, NH_4_^+^-N, NO_2_^−^-N, and NO_3_^−^-N concentrations of blank contrast aquarium **(A)** and the aquarium with the inbuilt MBBR **(B)**.

As illustrated in Figure 2A, the COD and NH_4_^+^-N concentrations of the blank contrast aquarium showed an upward trend over time due to the excretions of fishes and degradation of unconsumed feed (Hu et al., 2015; Zamora-Garcia et al., 2021). In addition, NO_2_^−^-N and NO_3_^−^-N concentrations were always below 0.6 mg/L, indicating less NH_4_^+^-N inverting to NO_2_^−^-N and NO_3_^−^-N. Especially on the 16th day, the NH_4_^+^-N concentration reached up to 1.985 mg/L with the NH_4_^+^-N production rate of 0.12 g/m^3^/d, and the fish did not tolerate it and began to die. At this point, the blank contrast aquarium experiment stopped.

The COD concentration of the aquarium with the inbuilt MBBR showed an upward trend over time during the first 5 days. Then it did not change with time, ranging from 0–25 mg/L (Figure 2B). At the same time, most carriers initially floating on the upper inside of the shell began to be suspended, indicating that microorganisms began accumulating after 5 days in the aquarium. Like COD concentration, the NH_4_^+^-N concentration also showed an upward trend over time during the first 5 days, began to decrease on the 6th day, and then reached 0.440 mg/L on the 9th day because of the fish food dosing. This concentration was also the maximum NH_4_^+^-N concentration during the experiment, less than a quarter of that in the blank contrast aquarium. And after that day, the NH_4_^+^-N concentration gradually decreased over time and went down to not be detected (<0.05 mg/L) on the 16th day. Then until the end of the experiment on the 90th day, the NH_4_^+^-N concentration was always below 0.05 mg/L, which could not threat the fish (Yildiz et al., 2017). It maybe because that NH_4_^+^-N was removed by ammonia oxidizing bacteria in the biofilms (Yildiz et al., 2017). NO_2_^−^-N concentration in this aquarium was always below 0.05 mg/L, indicating that NO_2_^−^-N maybe converted to NO_3_^−^-N by nitrate oxidizing bacteria (Yildiz et al., 2017). However, the NO_3_^−^-N concentration did not increase gradually, but fluctuated in the 0–4.5 mg/L range. There may be simultaneous nitrification and denitrification (SND) in the biofilms (Pan et al., 2022). Numerous studies have shown that MBBR was a well-established technology for SND, because the biofilms of a certain thickness, which provide a suitable micro environment (Liu et al., 2020; Bhattacharya and Mazumder, 2021). In conclusion, the inbuilt MBBR with suspended biofilms could be used for the nitrogen removal of household aquarium and performed well.

### 3.2 Nitrification performance of biofilms

**Figure 3:**
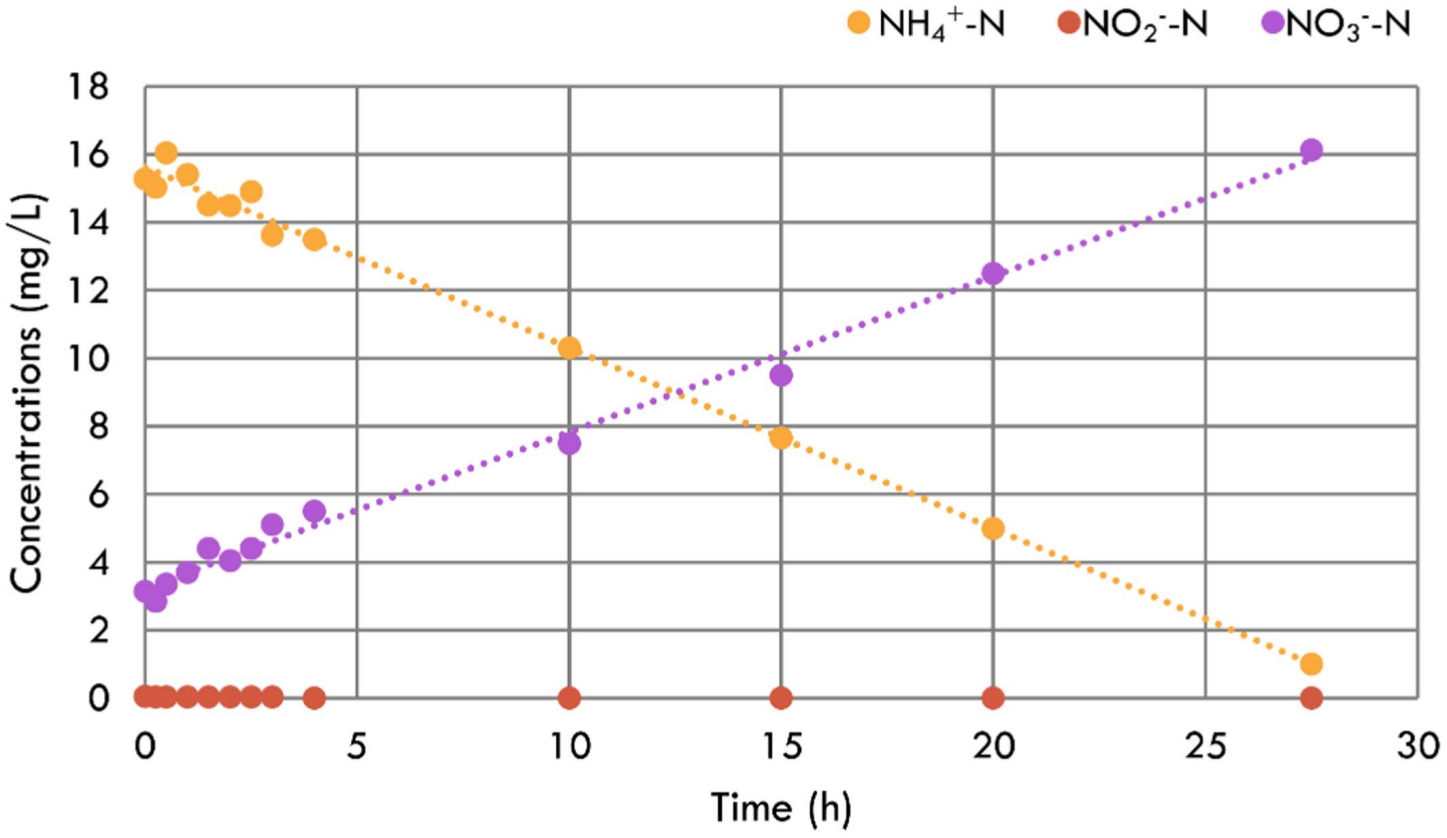
NH_4_^+^-N, NO_2_^−^-N, and NO_3_^−^-N concentration variations during the nitrification performance test for the suspended biofilms.

To explore the biofilm performance, NH_4_^+^-N, NO_2_^−^-N, and NO_3_^−^-N concentration variations were tested, and the results are shown in Figure 3. It was linearly fitted and calculated that the specific NH_4_^+^-N removal rate reached 45.43 g/m^3^/d based on the bio-carrier’s volume. According to the NH_4_^+^-N production rate of 0.12 g/m^3^/d, only 0.27% of suspended biofilms were needed in the aquarium. Given this, the inbuilt MBBR could be very small. Based on the bio-carrier’s specific surface area, the specific removal rate was 0.09 g/m^2^/d, which was lower than that applied in municipal wastewater (Zhou et al., 2022b)—the reason may be the limitation of the influent substrate (Han et al., 2022). During the test period, there was no accumulation of NO_2_^−^-N in the reactor, indicating that the nitrification was carried out thoroughly. At the same time, the NO_3_^−^-N concentration increased correspondingly due to the transformation of NH_4_^+^-N to NO_3_^−^-N by nitrification. Unlike the experimental operation in the aquarium, there was almost no NO_3_^−^-N removal in this reactor. The reason may be denitrification was restrained by the high DO concentration (Ren et al., 2022) or that the retention time of this test was shorter. In conclusion, the suspended biofilms performed a strong nitrification capacity, which could protect the fish from the NH_4_^+^-N.

### 3.3 Biofilm morphology

**Figure 4:**
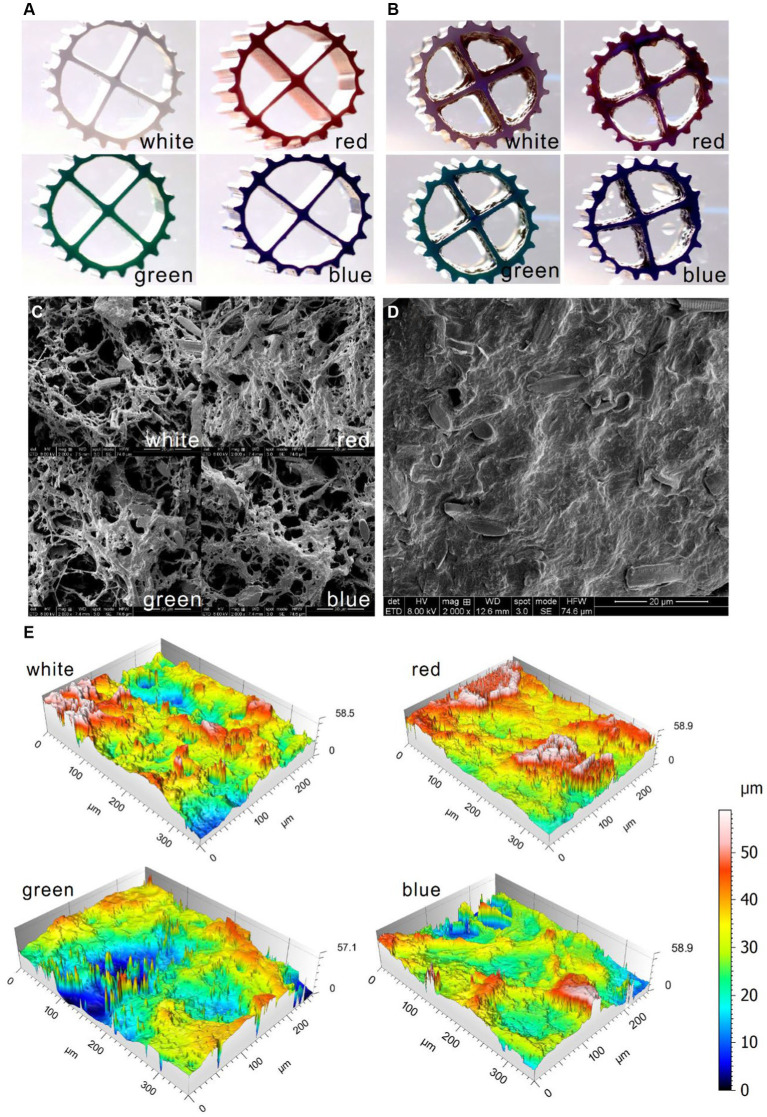
Morphology scanned by type microscope for the new carriers **(A)** and the bio-carriers in the aquarium **(B)**, by the SEM for the suspended biofilms **(C)** and the sediment **(D)** in the aquarium, and by the LSCM for the bio-carriers in the aquarium **(E)**.

The biofilm morphology in the aquarium was shown in Figure 4. Compared to the new carriers (Figure 4A), the biofilms attached to the carriers were clearly visible (Figure 4B). The biofilms presented a certain thickness, which was benefited for SND in MBBR (Bhattacharya and Mazumder, 2021). It was found that various forms of microorganisms and extracellular polymers were distributed in the suspended biofilms and the sediment by SEM magnification of 2,000 times (Figures 4C,D). The suspended biofilms and the sediment in the aquarium exhibited a considerable diversity of microorganisms, including coccus, bacillus, and hyphomycetes. Under the wash of oxygen, the suspended biofilms appeared sparse with many filamentous structures (Figure 4C), while the sediment in the aquarium was relatively dense (Figure 4D). The structure of the suspended biofilms is more beneficial to the transfer of substrate in microorganisms. There was little difference in the biofilm morphology among different colors of bio-carriers (Figure 4C). The construction of the inbuilt MBBR in the aquarium could help the suspended biofilm obtain oxygen and achieve a structure conducive to the transfer of substrate in microorganisms under the scouring of oxygen. According to the three-dimensional morphology (Figure 4E), the suspended biofilms attached to white, red, green, and blue carriers all showed a certain degree of roughness with the Ra values of 0.490, 0.809, 0.800, and 0.461 μm and the Rz values of 2.25, 5.59, 4.08, and 3.03 μm, indicating the formation of mature biofilms and efficient mass transfer of nutrients to biofilm (Wu et al., 2023). It could be seen that the suspended biofilms successfully cultured microorganisms in the household aquarium.

### 3.4 Bacterial community

**Figure 5:**
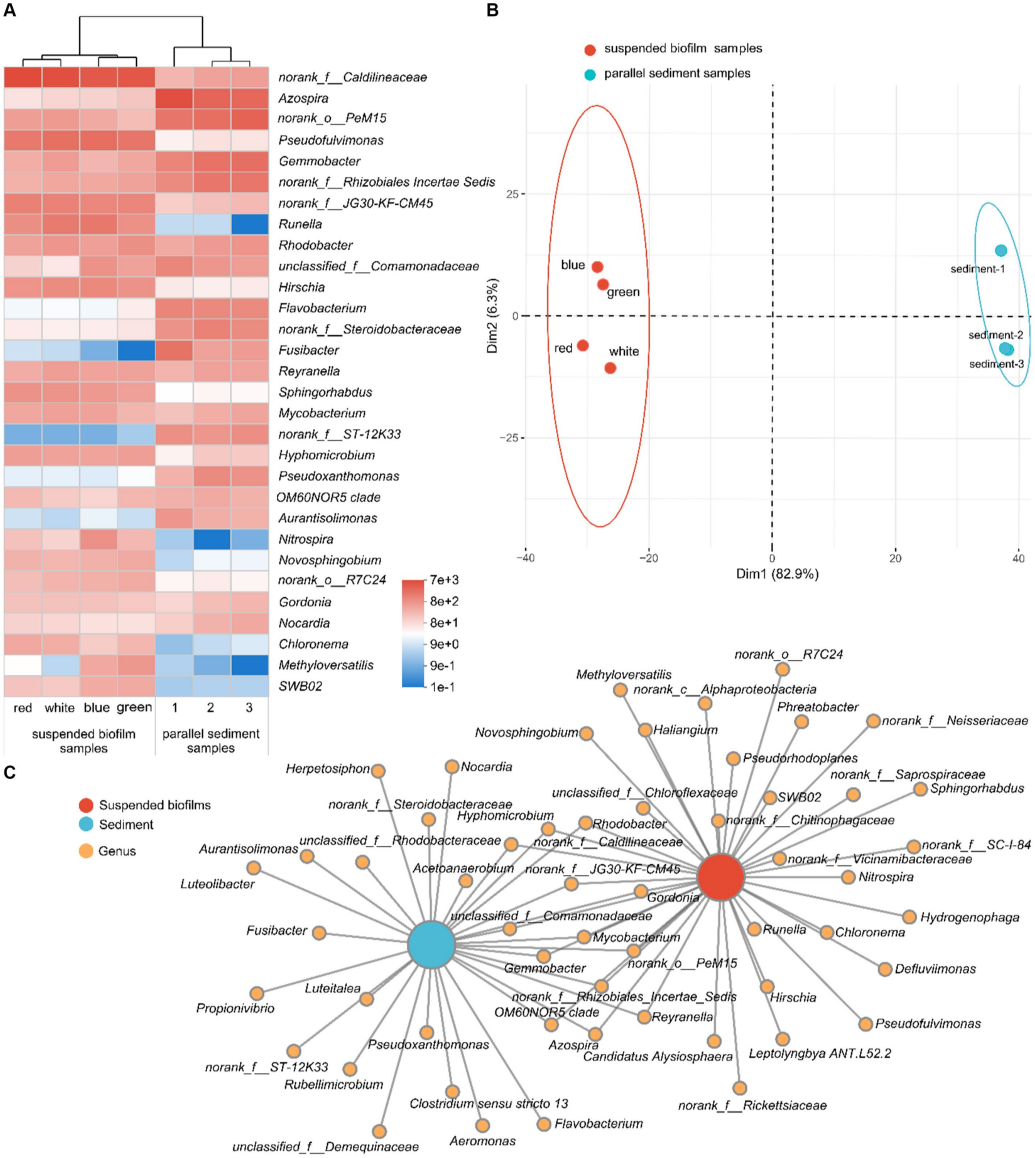
Heatmap for the relative abundance of the top 30 genera **(A)**; PCA for the bacterial community **(B)**; and collinear network analysis **(C)** of the suspended biofilms and sediment in the aquarium.

The bacterial communities of the suspended biofilms and sediment at the genus level are shown in Figure 5. As shown in the heatmap of relative abundance for the top 30 genera (Figure 5A), the seven samples were clustered into two groups, which were suspended biofilm samples and sediment samples. The PCA showed the same result, when PC1 and PC2 explained 82.9% and 6.3% variation, respectively (Figure 5B). The ANOSIM further confirmed that the grouping was statistically significant (R = 1.000, *p* < 0.05). In addition, there were 25 and 17 genera abundant (sequence number > 100) only in suspended biofilms and only in the sediment, respectively (Figure 5C). However, there were only 13 genera abundant (sequence number > 100) in both suspended biofilms and sediment. The above confirmed that the bacterial communities of suspended biofilms and sediment in the aquarium were quite differed.

As shown in Figure 5A, the most abundant genus in the aquarium was *norank_f__Caldilineaceae*, which belongs to the family Caldilineaceae. Caldilineaceae was reported to be highly related to domestic wastewater treatment system (Zhang et al., 2017) and was the critical active denitrifier in nitrogen removal (Cao et al., 2020). Correspondingly, denitrification occurred in the aquarium during the experimental operation. However, the average sequence number (relative abundance) of *norank_f__Caldilineaceae* in the biofilms and sediment was 5,706 (24.2%) and 434 (1.8%), respectively. The inbuilt MBBR enriched the genus *norank_f__Caldilineaceae*, which would benefit to denitrification in the aquarium. In addition to that, among the top 30 genus in the aquarium, the certain genera *Pseudofulvimonas* (Lu et al., 2023), *Gemmobacter* (Tan et al., 2020), *Runella* (Zhao et al., 2017), *Rhodobacter* (James et al., 2023), *Flavobacterium* (Fan et al., 2021), *Fusibacter* (Han et al., 2021), *Reyranella, Mycobacterium* (Jiao et al., 2022), *Hyphomicrobium* (Fan et al., 2021), *Pseudoxanthomonas* (Jian et al., 2023), *Novosphingobium* (Sun et al., 2022), *Gordonia* (Tran et al., 2022), and *Methyloversatilis* (Zhai et al., 2020) were all reported to be denitrifiers. *Pseudofulvimonas*, *Novosphingobium*, and *Methyloversatilis* were abundant (sequence number > 100) only in the suspended biofilms, while *Flavobacterium*, *Fusibacter*, and *Pseudoxanthomonas* were abundant only in the sediment (sequence number > 100; Figure 5C). In conclusion, there were a variety of denitrifiers in the aquarium, and the communities of the suspended biofilms and sediment were different. It explained the phenomenon of SND in the aquarium from a microscopic perspective.

The second most abundant genus was *Azospira*, which fixed nitrogen (Tan et al., 2003). This genus was abundant in both biofilms and sediment (sequence number > 100). However, the average sequence number (relative abundance) of *Azospira* in the biofilms and sediment was 108 (0.5%) and 4,111 (17.4%), respectively. Therefore, nitrogen fixation may occur in the aquarium. The third most abundant genus was *norank_o__PeM15*, which may come from the animal gut (Zhu et al., 2022). It is worth noting that nitrifying bacteria *Nitrospira* appeared in the aquarium, which was reported to be capable of complete ammonia oxidation to nitrate (Daims et al., 2015). *Nitrospira* was abundant (sequence number > 100) only in suspended biofilms, and the average sequence number (relative abundance) was 335 (1.4%). In sediment, the average sequence number of *Nitrospira* was only 1. The inbuilt MBBR with suspended biofilms enriched *Nitrospira*, which brought the function of nitrification to the aquarium. Correspondingly, nitrification occurred in the aquarium with the inbuilt MBBR but not in the blank contrast aquarium.

### 3.5 Potential functions for nitrogen removal

**Figure 6:**
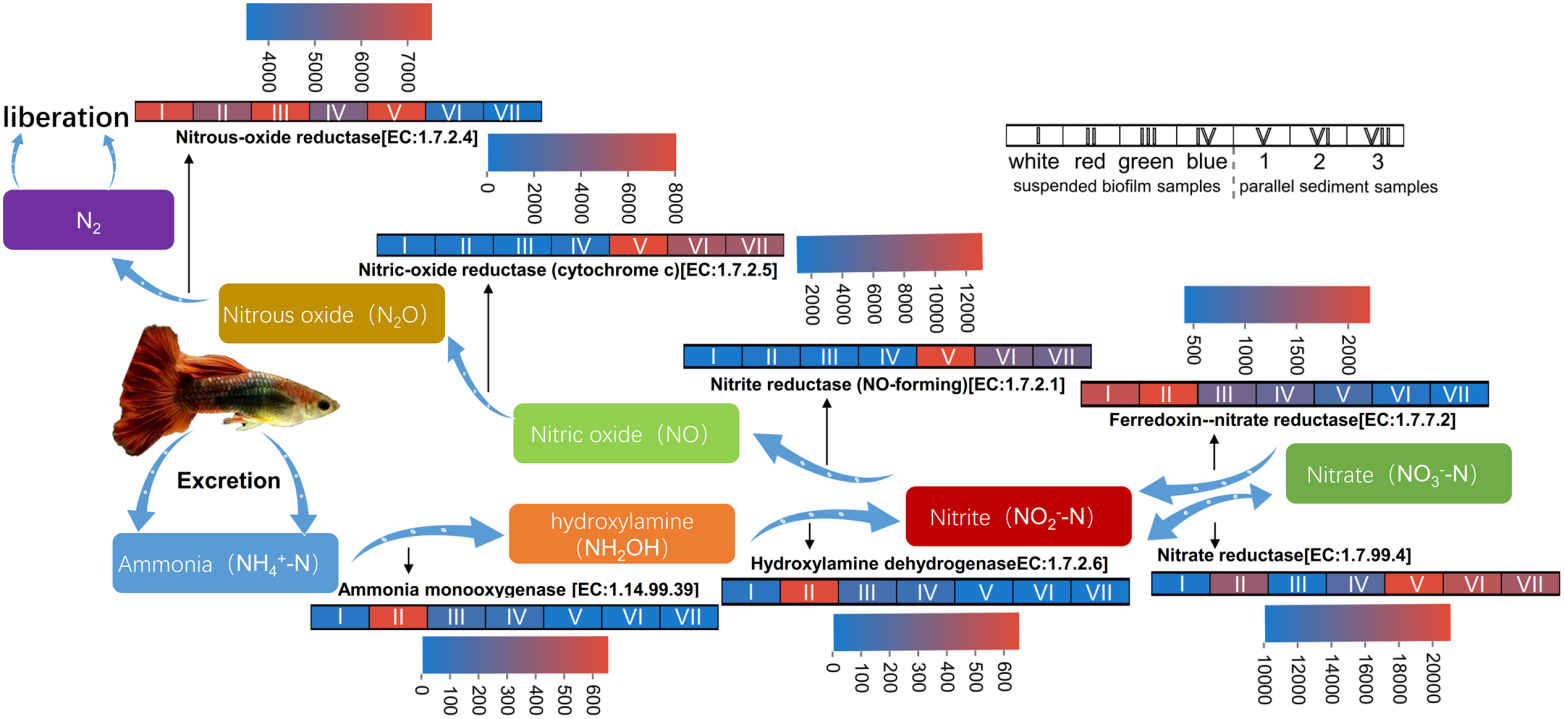
Heatmap for sequence numbers of the potential functions for nitrogen removal of the suspended biofilms and sediment in the aquarium.

The potential functions for nitrogen removal, including nitrification and denitrification, of the suspended biofilms and sediment in the aquarium are shown in Figure 6.

For the enzyme involved in denitrification, the nitrite oxidoreductase [EC:1.7.99.4] converts nitrate and nitrite into each other, and ferredoxin--nitrate reductase [EC:1.7.7.2] converts nitrate to nitrite (Zhou et al., 2022a). The average predicted sequence numbers of these two enzymes in suspended biofilms were 12,857 and 1,504, while the numbers in the sediment were 18,767 and 599. Nitrite reductase (NO-forming) [EC:1.7.2.1], nitric-oxide reductase (cytochrome c) [EC:1.7.2.5], and nitrous-oxide reductase [EC:1.7.2.4] converted nitrate to nitric oxide, then to nitrous oxide, then finally to gas nitrogen, respectively (Zhou et al., 2022a). The average predicted sequence numbers of these three enzymes in suspended biofilms were 1,675, 954, and 6,536, while the values in the sediment were 8,509, 6,421, and 5,152. So, there were certain sequences for these enzymes involved in denitrification in both suspended biofilm and sediment. It indicated that the suspended biofilms and sediment both had potentials in functions for denitrification. These conversion processes involved in denitrification are not restrictive steps for nitrogen removal in the aquarium.

After the fish ate, their excretion produced ammonia nitrogen, which harmed the fish. For the enzyme involved in nitrification, the ammonia nitrogen was converted to hydroxylamine by ammonia monooxygenase [1.14.99.39] and then to nitrate by hydroxylamine dehydrogenase [EC:1.7.2.6] in the aquarium (Zhou et al., 2022a). The average predicted sequence numbers of ammonia monooxygenase [1.14.99.39] in the suspended biofilms was 220, while it was only 5 in the sediment. The values of hydroxylamine dehydrogenase [EC:1.7.2.6] were 221 in the suspended biofilms, but it was only 1 in the sediment. It indicated that the suspended biofilms have the potential for the nitrification function. Therefore, the inbuilt MBBR played an essential role in ammonia nitrogen removal in the aquarium. Building an inbuilt MBBR in the household aquarium is feasible to protect the fish from nitrogen.

## 4 Conclusion

This study successfully established an innovative inbuilt MBBR in the household aquarium with the benefit of being small, feasible, and beautiful. It was shown that the ammonia nitrogen, nitrite nitrogen, and nitrate nitrogen concentrations were always below 0.5 mg/L, 0.05 mg/L, and 4.5 mg/L in the aquarium with inbuilt MBBR. There is SND in the aquarium with MBBR. The suspended biofilms presented a different morphology from the sediment, with a sparser structure and a rough surface. The bacterial community of the suspended biofilms also differed from the sediment. Denitrifiers were found in suspended biofilms and the sediment, but their communities differed. What was essential was that the suspended biofilms in MBBR enriched nitrifying genus *Nitrospira* and had a potential function of ammonia monooxygenase [1.14.99.39] and ammonia monooxygenase [1.14.99.39], which was different from the sediment. Therefore, this research proved that the introduction of MBBR to household aquariums is feasible, which provides a new idea for nitrogen removal in the household aquarium. It also provided a scientific basis for the design and application of MBBR in household aquariums.

## Data availability statement

The datasets presented in this study can be found in online repositories. The names of the repository/repositories and accession number(s) can be found in the article/supplementary material.

## Ethics statement

The manuscript presents research on animals that do not require ethical approval for their study.

## Author contributions

XZ: Conceptualization, Data curation, Formal analysis, Investigation, Methodology, Visualization, Writing – original draft, Writing – review & editing. HL: Data curation, Formal analysis, Investigation, Writing – original draft. XF: Data curation, Investigation, Validation, Writing – original draft. XX: Data curation, Formal analysis, Investigation, Writing – original draft. YG: Data curation, Investigation, Writing – original draft. XB: Conceptualization, Funding acquisition, Project administration, Resources, Supervision, Validation, Writing – review & editing. LC: Conceptualization, Supervision, Validation, Writing – review & editing. SH: Conceptualization, Funding acquisition, Supervision, Validation, Writing – review & editing. FZ: Supervision, Validation, Writing – review & editing. TY: Supervision, Validation, Visualization, Writing – review & editing.

## Footnotes

^1^ https://www.arb-silva.de/

^2^ www.majorbio.com

^3^ https://www.kegg.jp/pathway/map00910
